# Hybridization following population collapse in a critically endangered antelope

**DOI:** 10.1038/srep18788

**Published:** 2016-01-06

**Authors:** Pedro Vaz Pinto, Pedro Beja, Nuno Ferrand, Raquel Godinho

**Affiliations:** 1 CIBIO/InBio - Centro de Investigação em Biodiversidade e Recursos Genéticos, Universidade do Porto, Campus Agrário de Vairão, 4485-661 Vairão, and Departamento de Biologia, Faculdade de Ciências, Universidade do Porto, Rua do Campo Alegre s⁄n. 4169-007 Porto, Portugal; 2 ISCED – Instituto Superior de Ciências da Educação da Huíla, Rua Sarmento Rodrigues, Lubango, Angola; 3 Fundação Kissama, Rua Joaquim Capango n°49, 1°D, Luanda, Angola; 4 Department of Zoology, Faculty of Sciences, University of Johannesburg, Auckland Park 2006, South Africa

## Abstract

Population declines may promote interspecific hybridization due to the shortage of conspecific mates (Hubb’s ‘desperation’ hypothesis), thus greatly increasing the risk of species extinction. Yet, confirming this process in the wild has proved elusive. Here we combine camera-trapping and molecular surveys over seven years to document demographic processes associated with introgressive hybridization between the critically endangered giant sable antelope (*Hippotragus niger variani*), and the naturally sympatric roan antelope (*H. equinus*). Hybrids with intermediate phenotypes, including backcrosses with roan, were confirmed in one of the two remnant giant sable populations. Hybridization followed population depletion of both species due to severe wartime poaching. In the absence of mature sable males, a mixed herd of sable females and hybrids formed and grew progressively over time. To prevent further hybridization and recover this small population, all sable females were confined to a large enclosure, to which sables from the other remnant population were translocated. Given the large scale declines in many animal populations, hybridization and introgression associated with the scarcity of conspecific mates may be an increasing cause of biodiversity conservation concern. In these circumstances, the early detection of hybrids should be a priority in the conservation management of small populations.

Human-mediated hybridization is increasing worldwide, and contributes to species extinctions through direct and indirect means^1^,^2^,^3^. In mammals, anthropogenic hybridization often results from interbreeding between domestic species and their wild relatives^4^,^5^,^6^, but it may also involve related *taxa* that are artificially managed or introduced outside their natural range^7^,^8^,^9^. Hybridization between naturally sympatric species is much less common, though it may occur when a rare species interbreeds with a common species due to the shortage of conspecific mates^10^,^11^,^12^,^13^, in what is often referred to as the Hubb’s principle or “desperation hypothesis”^14^. Confirmation of this hypothesis in wild populations, however, is hindered by difficulties in detecting the moment when reproductive isolation is disrupted and identifying its underlying demographic causes. Yet, further testing this idea and evaluating its consequences for biodiversity conservation is important due to the complexity of policies and management decisions involving hybrids^2^,^15^,^16^,^17^.

The giant sable is a distinct monophyletic group confined to central Angola, and isolated from much larger sable populations living elsewhere in eastern and southern Africa^18^ (Fig. 1). Giant sables are critically endangered^19^, with a small natural range comprising only the protected areas of Cangandala National Park (hereafter Cangandala) and Luando Integral Nature Reserve (hereafter Luando)^20^. The subspecies was feared extinct for over a decade, but it was rediscovered in Cangandala in 2005, where preliminary evidence suggested that they had suffered a marked decline from historical levels during the Angolan civil war (1975–2002)^21^. Ensuing camera-trap monitoring to determine population status allowed the detection of individuals with unusual morphological features, which led to the suspicion that hybridization could be occurring between giant sable and roan antelopes^22^. The possibility of hybridization between these two phenotypically distinct antelopes was unexpected because they have evolved in sympatry for a relatively long time^23^, following divergence from the most recent common ancestor estimated to have occurred in the late Miocene^24^,^25^. Therefore, both species were expected to have developed efficient barriers that prevent interbreeding in most situations^26^. Nevertheless, a single case of hybridization had already been reported from the Kruger National Park in South Africa^26^.

Given the conservation implications of these initial results, we started a study combining field and molecular methods (see Methods) to (1) census the population and estimate demographic trends; (2) confirm hybridization and introgression events; (3) identify maternal and paternal ancestry of hybrids; and (4) estimate the demographic processes underlying the putative hybridization events. The intensive monitoring of this population over seven years provided a unique opportunity to test Hubb’s desperation hypothesis, by evaluating whether the putative hybridization events could be linked to the shortage of conspecific mates. Results from this study were also used to trigger emergency conservation action to avoid further hybridization and promote population recovery.

## Results

Intensive camera-trapping revealed that shortly after the end of the Angolan civil war only one giant sable herd was present in Cangandala (Supplementary Fig. 1). In 2005, the herd comprised adult females, calves, and young animals estimated to have been born within the previous three years (Fig. 2), all of which displayed the typical morphological features of giant sables (Fig. 3). The herd also included calves and young individuals with phenotypic features intermediate between sable and roan, the oldest of which were estimated to have been born in 2002. These individuals could be distinguished from sable by their larger size, paler mane coloration, and differences in facial mask and ear shape (Fig. 3). The number of roan detections through camera-trapping was nil in 2005 but then increased over time (Supplementary Table 1), though most records until the end of 2008 corresponded to a single juvenile female (2006) and two solitary males (2006–2008). The first roan herd was recorded only in the second half of 2008, and then the number of herd detections increased during 2009–2011.

Births of phenotypically pure sable calves were not observed after 2005 and were only recorded again in 2010, following the translocation to Cangandala of a sable bull captured in Luando. Births of putative hybrid calves were estimated to have occurred every year since 2002, but they were not detected again after 2010 when all phenotypically pure female sables were physically separated from putative hybrids and roan males. The number of putative hybrids recorded grew steadily over time, equalling sables in 2009 (Fig. 2). Two individuals born in 2007 and 2009 appeared morphologically different from the other putative hybrids, as they showed features more closely resembling roan (Fig. 3). In contrast, the last putative hybrid born in 2010 resembled sable. The presence of these three calves, plus the observation of a putative hybrid female with udders and attending two of them in 2009 and 2010 (Supplementary Fig. 1e), strongly suggested the occurrence of second generation hybrids. No mature sable bull indigenous to Cangandala was ever observed throughout the study, though a young male was present in 2005 but disappeared thereafter. Four sable male calves born in 2005 also disappeared subsequently before maturing. Between 2005 and 2009 the herd was dominated in succession by three different putative hybrid bulls, and was often in close proximity to a solitary roan bull.

Genetic diversity based on microsatellites was significantly lower (p < 0.05) in giant sables from Angola (2.47 alleles per locus; H_E_ = 0.329; N = 35), compared to Namibian sables (3.41 alleles per locus; H_E_ = 0.450; N = 22), and particularly roan (5.63 alleles per locus; H_E_ = 0.488; N = 24) (Supplementary Table 3). Divergence between Angolan and Namibian sables was statistically significant (F_ST_ = 0.438, p < 0.001), and between these and roan (F_ST_ = 0.523 and F_ST_ = 0.467, respectively, p < 0.001). In the biplot of a Principal Components Analysis (29.0% of overall genetic variation), there were three clear clusters corresponding to Angolan and Namibian sables, and roan, whereas the nine putative hybrids genotyped from Cangandala showed an intermediate position between roan and giant sable (Fig. 4a).

STRUCTURE analysis for K = 2 clearly differentiated sable from roan (Fig. 4b), with mean posterior probability of individual assignment to correct species of q_i_>0.99. All morphologically intermediate individuals were partially assigned to both species, with individual q_i_ ranging from 0.21 to 0.79. Bayesian clustering analysis to detail hybrids ancestry ascribed all putative hybrids to strict hybrid classes with posterior probabilities above 99%, including the confirmation of two individuals as backcrosses to roan (Supplementary Table 2). All hybrids had the giant sable mitochondrial haplotype previously identified in Cangandala^21^, and the Y chromosome of the three hybrid males was typical of roan (Supplementary Table 2). In maternity analysis, each of the seven F1 hybrids analysed were ascribed to one of four sable females with a strict confidence level of 99% (Supplementary Table 2). The two backcrosses were assigned to a single hybrid female (Supplementary Table 2). The results of paternity analysis were compatible to the view that a single roan bull sired all hybrids (Supplementary Table 2), but this could not be further explored due to the few genetic data for Angolan roans.

## Discussion

Our results confirmed the occurrence of introgressive hybridization between giant sable and roan antelopes, which was associated with the presence of a solitary male roan in the home range of an isolated sable herd, at least temporarily unattended by mature sable bulls. Individuals with hybrid morphology had never been reported before in Cangandala, though this population was well studied in the 1970s, when both sable and roan were common in the area^20^. In contrast, during our study we found a single sable herd in Cangandala, whereas roan herds appeared to be absent until the end of 2008. Population depletion of both species was probably a consequence of heavy wartime poaching since at least the early 1980s, which may well have intensified after the war ceased in 2002. Evidence of illegal hunting during our study included encounters with poachers, apprehension of shotguns and AK-47’s, finding and dismantling of hundreds of traps, and severe leg wounds caused by snares observed in several sables and roans handled. As observed in other African antelopes^27^,^28^, poaching possibly had a particularly strong effect on sable males, because their contrasting coloration, solitary behaviour and long-range movements may make them more vulnerable to poachers than females. Systematic elimination of sable bulls may have resulted in the lack of conspecific mates for sable females, thus favouring heterospecific mating with a male roan. Although the birth of pure sables between 2002 and 2005 suggested that mature male sables were still in the area, these may have been young and inexperienced individuals with a weak control of the herd, which were presumably killed by poachers. This idea is supported by the detection on two occasions in 2005 of a young adult (about 2.5 years old) sable male accompanying the herd, which soon disappeared. Mature roan females appeared very scarce or even absent in Cangandala before 2008, and so the shortage of conspecific mates could also have promoted the initial heterospecific mating by the male roan.

Karyological uniformity of sable and roan antelopes may have facilitated the production of viable hybrids^26^, suggesting that current reproductive isolation between the two species is maintained mainly via prezygotic barriers. However, there was some evidence for reduced viability and fertility of hybrids, though results should be interpreted with caution due to small sample sizes. First, there was a tendency for more females (≈70%) than males in the group of confirmed F1 hybrids (n = 7), which is in line with the reduced viability for the heterogametic sex predicted by Haldane’s rule. Second, hybrid males appeared to be sterile, as no offspring was produced by the two confirmed and one putative hybrid bull that had access to breeding sable and F1 females for several years in succession. Third, reduced fertility was also apparent in F1 females, as only one out of five showed signs of pregnancy and produced calves. Finally, fitness of backcrosses was unknown, but is noteworthy than one of the two roan backcrosses had physical abnormalities, while the suspected sable backcross died at young age for unknown reasons. It is unlikely, therefore, that this mixed herd of sable females, F1 and backrosses would develop into a hybrid swarm with long-term viability^1^,^2^. Instead, it is likely that without conservation intervention this hybridization event would represent a dead end for the local sable population, culminating a long term population decline. Nevertheless we can’t exclude the possibility that some sable genes could eventually introgress further into the local roan population.

Although there is increasing recognition of the evolutionary and ecological roles of hybrids^15^,^16^, human-mediated hybridization is considered undesirable and something to be eliminated if possible^1^,^2^. If a population has not become a hybrid swarm and still contains a number of parental individuals, management recommendations include removal of hybrids or captive-breeding programs^2^. In our case, conservation action was taken in 2009–2011, when all surviving hybrids and sable females were captured and confined in large enclosures, and three sable bulls and six adult females from Luando were translocated into Cangandala (Supplementary Fig. 2). The decision to intervene was taken because the local population was considered technically extinct, with a single herd including nine old pure females and several F1 and backcross hybrids, with low genetic diversity and no prospects for a male sable to be naturally recruited into the population. Birth of three sables from two old females that previously mothered hybrids occurred in 2010–2011, providing the first signs of population recovery. To the best of our knowledge this is the first example of successful management action taken in response to the detection of introgressive hybridization in the wild between two naturally sympatric species, reinforcing the importance of early detection of admixed individuals in conservation programs of endangered species^4^,^5^,^13^.

By catching hybridization in flagrante delicto, this study provided support for the operation in the wild of Hubb’s ‘desperation’ hypothesis^14^, though in our case interbreeding occurred between two rare species, rather than between a rare and a common species. Other studies have reported hybridization events between naturally sympatric species that are compatible with Hubb’s principle, though with limited information on the underlying demographic processes. For instance, a population decline in harbor porpoises *Phocoena phocoena* was hypothesised to have resulted in reduced male discrimination of mates and hybridization with Dall’s porpoises *Phocoenoides dalli*^10^; hybridization in African cercopithecine monkeys was associated with habitat fragmentation and small population sizes^29^; local extinction due to overexploitation followed by recolonization was considered to have resulted in hybridization among three species of fur seal *Arctocephalus* spp.^11^; males of the endangered Grevy’s zebra *Equus grevyi* at the edge of the species range were hypothesised to have difficulties in finding conspecifc mates and thus to interbreed with locally abundant plain’s zebra *Equus quagga* females^12^. In all these cases, evidence suggest that hybridization was preceded by reductions in population size or isolation/expansion events on local populations, which resulted in the scarcity of conspecific mates. Considering that severe population declines and fragmentation processes are affecting an increasing number of species^30^, hybridization and introgression associated with the scarcity of conspecific mates may be an increasing cause of biodiversity conservation concern. Addressing this problem should involve efforts for the early detection of hybrids in small populations, which could then prompt rapid conservation management action.

## Methods

### Study design

**Figure 1:**
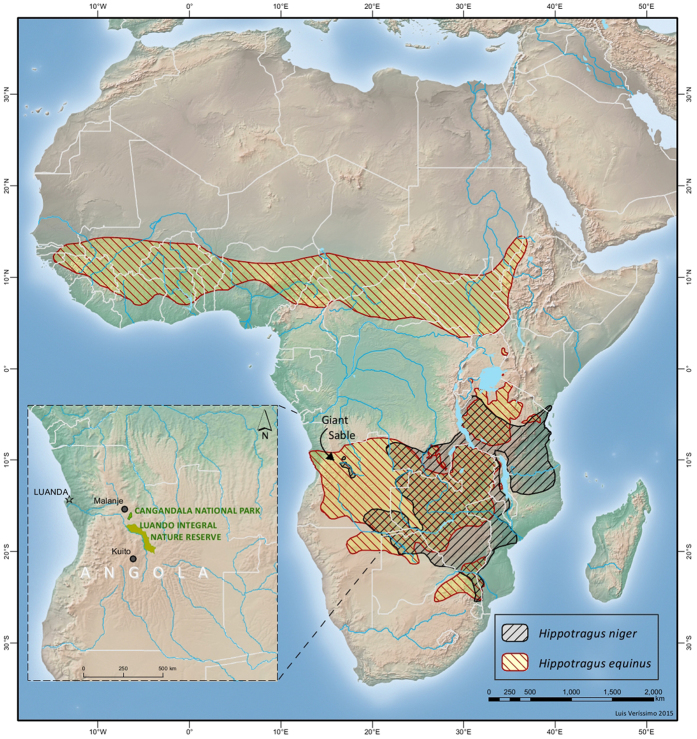
Distribution of sable (*Hippotragus niger*) and roan (*H.* equinus) antelopes in Africa and location of the study area. The giant sable antelope (*H. niger variani*) has a very small range, which is far apart from the species core distribution in eastern Africa. Giant sables only occur in Angola (inset), where they are restricted to the Cangandala National Park and the Luando Integral Nature Reserve. Geographic ranges are adapted from IUCN (2008a. *Hippotragus niger*. The IUCN Red List of Threatened Species. Version 2014.3) and IUCN (2008b. *Hippotragus equinus*. The IUCN Red List of Threatened Species. Version 2014.3). The Figure was produced by Luís Verissimo.

**Figure 2:**
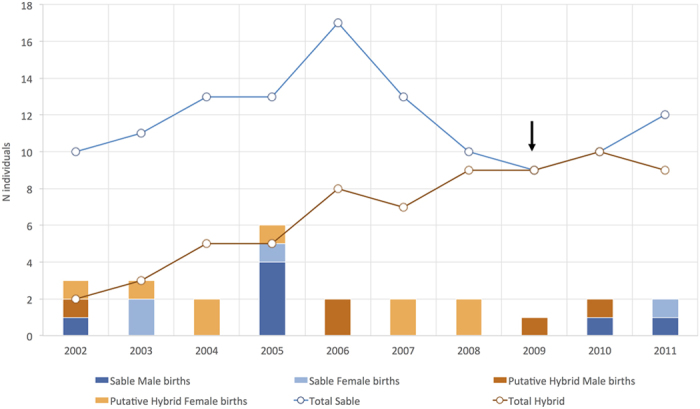
Temporal variation in estimated population size and births of giant sable antelopes and sable x roan hybrids in Cangandala National Park, Angola. The number of hybrids recorded grew progressively over time (red line), while the number of sable observed increased up to 2006 and declined thereafter (blue line). We recorded births of male (dark blue bars) and female sables (light blue bars) between 2002 and 2005, and then again in 2010–2011, when all sables in Cangandala were taken to a large outdoor enclosure, to where sables captured in Luando National Park were translocated. Male (dark orange bars) and female (light orange bars) putative hybrids were estimated to be born each year until 2010, when access of roan to sable females was prevented. Arrow indicates the first introduction of a giant sable bull translocated from the Luando Integral Nature Reserve.

The study was based on intensive surveys of the giant sable antelope in Cangandala National Park (North-central Angola, Fig. 1), following the rediscovery therein of a remnant population that had been feared extinct^21^. Cangandala (>1,000 m a.s.l.) covers roughly 63,000 ha of gently undulating terrain, with nutrient-poor and extremely leached sandy soils, and dominated by dense and mature *Brachystegia* woodlands, interspersed by grasslands along a few drainage lines. A formal sampling procedure could not be established to survey sables, due to difficulties of regularly accessing this remote region and the extremely hard logistic conditions to develop field work, particularly in the first years after the end of the civil war. To overcome these problems, we used a combination of camera-trapping, aerial surveys, and molecular methods, aiming to census all the animals present in the region. Surveys benefited from the behaviour displayed by sables and other herbivores in Cangandala of regularly visiting some old termite mounds that the animals used as natural salt licks^31^, and where they could be predictably recorded. At the end of the study period, intensive efforts were developed to capture and confine in enclosures all phenotypically pure sables and putative hybrids, providing support to the results of censuses carried out in previous years. Tissue samples from animals captured were used in genetic analysis. This study was approved by the Unidade de Gestão e Coodenação da Estratégia da Biodiversidade, of the former Ministério do Urbanismo e Ambiente of Angola, and was carried out in accordance with the approved guidelines.

### Field methods

Between January 2005 and August 2011 we set camera traps in 18 sites, corresponding to natural salt licks (termite mounds) that were suspected to be visited by giant sables. Sites were identified with the help of local rangers, based on the presence of spoor and witness accounts of visual observations. Due to access and logistic difficulties, the number and type of cameras, as well as their operating period, varied greatly in space and over time. Until the end of 2007 we used six TrailMaster infrared trail monitors TM1500 and TM1550 connected to external photography film cameras or digital video. In October 2007 we introduced up to 12 passive digital cameras Stealth Prowler DVIR5. Due to variation in sampling effort and demographic changes, the number of photos recorded for sable and putative hybrids varied greatly from <25 (2005) to >9,000 (2010) (Supplementary Table 1). The large number of photos taken distributed over many independent events (i.e., visits by herd or individuals to salt licks in different days) (Supplementary Table 1), allowed a detailed recording of all animals and their individual identification (see below).

In July and August of 2009 and 2011, we conducted extensive aerial surveys and capture sessions that involved darting animals from a helicopter, aiming to capture all sables and putative hybrids remaining in Cangandala. The aerial surveys were maintained for several days after the last animal had been spotted and captured in 2011. Although dense canopy cover in most of the area makes it impossible to guarantee that all animals were indeed captured, it is very unlikely that any giant sable or hybrid remaining outside enclosures would be missed, as the Cangandala region is relatively small and has been visited regularly during field work carried out to the present day.

### Population estimates

**Figure 3:**
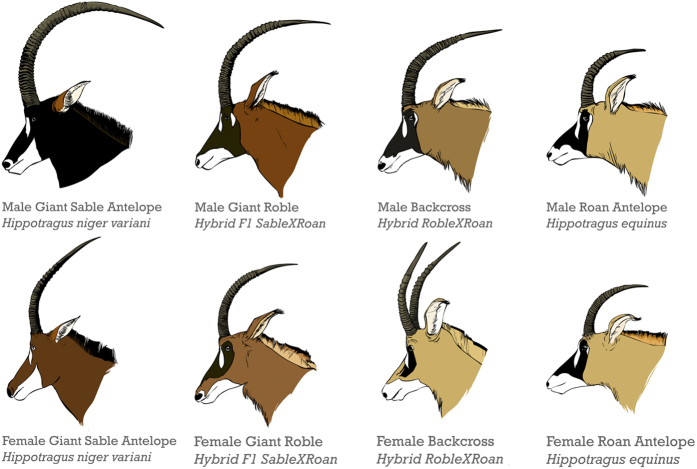
Schematic representation of diagnostic field characteristics of giant sable antelopes, roan antelopes, and their F1 hybrids and backcrosses observed in Cangandala National park, Angola. Drawings are based on photographs taken during camera trapping and animal capture sessions carried out in 2009–2011. The hybrids and backcrosses represented were confirmed through genetic analysis. The sable x roan hybrids (robles) are phenotypically intermediate between the parental species, while the hybrids x roan backcrosses have features more closely resembling roan. The female backcross shows the abnormal horns observed in a single individual.

**Figure 4:**
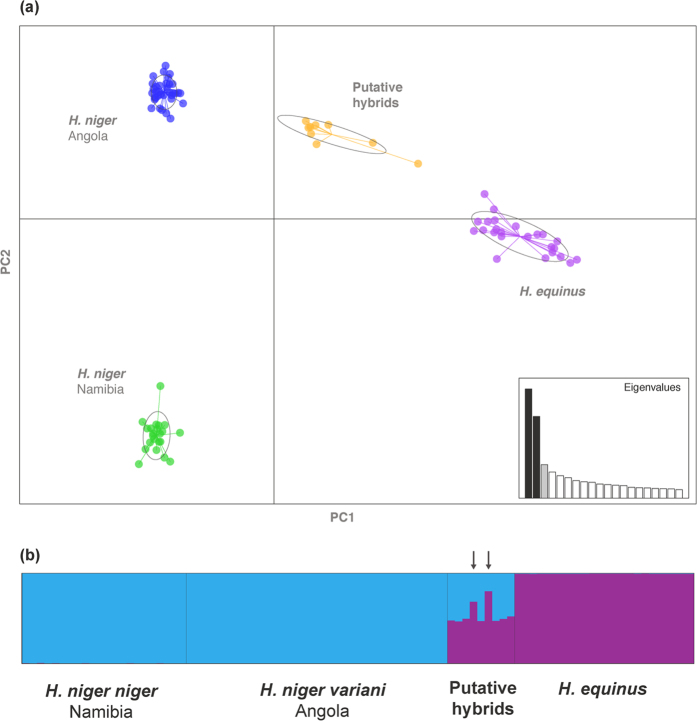
Genetic evidence for sable x roan hybridization in Cangandala National Park, Angola. **(a)** First and second components of a principal components analysis (PCA) of 51 microsatellite genotypes from 90 *Hippotragus* spp. samples; ovals are 95% inertia ellipses; the inset shows the distribution of eigenvalues for all principal components. **(b)** Individual assignment to genetic clusters (K = 2) inferred by Bayesian analyses (STRUCTURE); arrows indicate first generation backcrosses to roan confirmed by NewHybrids analysis. Phenotypically intermediate individuals are located between roan and sable in the PCA biplot, and they are partially assigned to both species in the STRUCTURE analysis.

Each year, the minimum number of sable, roan and putative hybrids present in Cangandala was estimated from the number of individually recognizable animals detected in photos and videos obtained through camera-trapping. Assignment of individuals to gender and age class further allowed the estimate of sex-ratio, age structure and annual number of births. To obtain these estimates, we first screened all photos and videos for the presence of sable, roan and putative hybrids. Each animal recorded in a photo or video was then identified to species-level, and ascribed to gender and age class. Species identification was straightforward, given the marked morphological differences between sable and roan. The individuals with intermediate morphology, or showing unusual phenotypes, were classified as putative hybrids. (Fig. 3). Gender was determined based on sexually dimorphism features displayed by both species. Approximate age was estimated from body size and horn development (size and number of natural annulations) of individuals; horns are useful in age determination because they show continuous growth throughout the life of sables^32^. In Cangandala we found that sable consistently developed seven horn annulations per year and these could be reliably counted from photographs in animals at least up to the age of four, when the annulations grew thinner and become increasingly harder to discriminate. Using these characteristics and by following the same animals over an extended period, from juvenile to maturity, we are confident that estimates of age class were accurate for animals under five years old. Animals were also identified individually based on a combination of species, gender, age and unique morphological features. In particular, we noted differences in facial mask pattern, including presence or absence of muzzle stripe and the extent of black and white patches. Occasionally there were horn abnormalities and other particular features that aided in individual identification. Individual identification of roan was difficult because animals are apparently less distinctive, and so roan population size in Cangandala could not be accurately estimated when more animals were recorded in 2009–2011.

The annual number of births of sable and putative hybrids was estimated by combining information on females with evidence of pregnancy, and the number and estimated age of calves, juveniles and young adults. Specifically, calves were assumed to be born either in the year when they were first observed or in the previous year, based on the timing of observations, body size and horn development^32^. Individuals <5 years old detected at the beginning of the study (2005–2006) were ascribed whenever possible to an approximate birth year, based on their estimated age^32^. Estimating the year of birth in Cangandala was made harder because calving occurred throughout the year, rather than showing the well-defined birth season in May-June as reported in studies carried out in the 1970s^20^. Also, the number of births each year should be taken as minimum estimates, because at least some individuals may have been born in 2002–2004 but died before camera-trapping started in 2005. It is not possible to exclude the possibility of a new-born dying before being recruited into the herd and subsequently photographed, even during the periods when camera-trapping was most intensive. Despite these problems, we believe that the minimum annual birth estimates presented in our study are sufficiently accurate to illustrate the temporal variation in the annual production of pure sable and hybrid calves by the population between 2002–2011.

The information obtained through camera trapping was complemented by aerial surveys and the capture sessions carried out in 2009 and 2011. Aerial surveys confirmed that a single sable herd was present in Cangandala during the entire study period, and the number of individuals spotted was compatible with camera-trapping data. Also, we never recorded a mature sable male during the aerial surveys, which matches the results from camera trapping. This was in contrast with similar surveys carried out in Luando, where a comparable sampling effort allowed the detection of 16 mature males and at least 4 herds. The animals captured in 2009 and 2011 in Cangandala, totalling nine giant sable females and nine hybrids, were the same recorded through camera trapping in previous years. No new animals were found, and we confirmed the individual identifications previously made using facial mask patterns and other unique features. Overall, close matching between data obtained through camera trapping, aerial surveys and capture sessions suggest that population parameters were accurately estimated.

### Genetic samples

During the capture operations carried out in 2009 and 2011, we collected 18 ear tissue samples, representing the whole lot of giant sable and putative hybrids surviving in Cangandala. During the concurrent capture operations carried out in Luando we collected ear tissue samples from another 26 giant sables and four roans. To allow comparisons with other sable and roan populations, we obtained 22 sable samples collected in Etosha National Park and Waterberg National Park in Namibia, both descendent from an original population translocated from the Caprivi Strip of Namibia^33^. We also obtained 20 roan samples collected in Caprivi (n = 5) Namibia, and in Percy Fyfe Nature Reserve (n = 2), Nylsvlei Nature Reserve (n = 11) and Marakele National Park (n = 2) in South Africa. The South African roans are presumed to be a mixed gene pool between indigenous animals and roans translocated from Namibia and Botswana^34^.

### Molecular procedures

Genomic DNA was extracted from tissue samples using the DNeasy Blood & Tissue Kit (QIAGEN), following manufacturer instructions. Individual multilocus genotypes were scored using a set of 51 sable-specific microsatellites, which all proved to be polymorphic in one or both species^35^. Amplification of loci followed the methodology and conditions as in Vaz Pinto *et al.*^35^, always using negative controls to monitor for possible contaminants. PCR products were separated by size on an ABI3130xl Genetic Analyzer. Allele sizes were scored against the GeneScan 500 LIZ Size Standard, using GENEMAPPER 4.0 (Applied Biosystems) and manually checked twice. The accuracy of genotypes was confirmed through re-amplification and re-analysis of 20% of random selected samples for each locus^36^, resulting in complete concordance among replicates. The microsatellite data were deposited in the Dryad Repository: http://dx.doi.org/10.5061/dryad.g615h.

To address the direction of hybridization, all individuals were scored for mitochondrial (mtDNA) lineage using primers LS-1 (5′-AATATACTGGTCTTGTAAACC [15305]) and HS-3 (5′-AGGCATTTTCAGTGCCTTGC [20]) to amplify 1100bp of the mtDNA control region^37^. Numbers in square brackets after the primer refer to the 5 ′ position of the primer, as localized on the nucleotide sequence of the *Hippotragus niger* complete mitochondrial DNA (KM245339^38^). Additionally, males were scored for the Y-linked haplotype using primers FY1 (5′-AAACAGTGCAGTCGTATGCTTCTGC) and RY1 (5′-GCCTTTGTTAGCGAGAGTAAGGAAG) to amplify 690bp of the SRY gene^39^. Amplifications were performed using the MyTaq™ Mix (Bioline) following manufacturer’s instructions, 0.5 μM of each primer and approximately 10 ng of genomic DNA in a total volume of 10 μl. PCR was performed over 38 cycles with the annealing temperature set to 56 °C (mtDNA) or using a touchdown profile with 13 cycles of annealing temperature of 58 °C, decreasing 0.5 °C per cycle, followed by 27 cycles with annealing temperature set to 52 °C (SRY). Successful amplifications for mtDNA and SRY were sequenced for both strands following the BigDye Terminator v3.1 Cycle sequencing protocol (Applied Biossystems) and sequenced in an ABI 3130xl Genetic Analyzer. Sequences were aligned using SEQSCAPE 2.5 (Applied Biosystems) and checked manually. mtDNA and SRY sequences were deposited in GenBank (Accession numbers KU146571 to KU146574).

### Molecular data analysis

Microsatellite diversity was evaluated separately for sable from Angola and Namibia, and roan, excluding putative hybrids, based on mean number of alleles per locus (Na) and observed (H_O_) and expected (H_E_) heterozygosities for each locus/population using GENETIX 4.05^40^. The same software was used to evaluate deviations from Hardy–Weinberg equilibrium. To test pairwise linkage disequilibrium between genotypes for all loci we used FSTAT 2.9.3.2^41^. Significance levels were adjusted using the sequential method of Bonferroni for multiple comparisons in the same data set^42^. Population differentiation was assessed by Fisher’s analogues of pairwise mean F_ST_ (estimator θ^43^) using also FSTAT. This software was also used to perform pairwise tests of differentiation for populations using the significance value of p < 0.001.

The occurrence of admixture among the nine putative hybrid individuals was investigated using the model-based Bayesian clustering analysis implemented in STRUCTURE 2.3.4^44^,^45^. The 90 individuals in the dataset were assigned to two populations (K = 2) without any prior for population identification and using the admixture model with correlated allele frequencies. The analysis was performed 10 times for 10^6^ MCMC steps following a burn-in period of 10^5^ steps. Posterior probabilities of the data in each run were compared to ascertain confidence in the model fit. To corroborate inferences from the STRUCTURE analysis without making assumptions regarding data structure, we used a centred, scaled PCA to cluster individual microsatellite genotypes. PCA clusters individuals only based on genotypes and has no assumptions regarding Hardy–Weinberg or linkage equilibrium. The PCA was conducted using adegenet package^46^ in R 3.1.2^47^. Additionally, NEWHYBRIDS 1.1^48^ was used to achieve a more detailed analysis of the hybrids’ ancestry, by inferring the posterior probability assignment (q) of each individual to six genotype frequency classes: sable, roan, F1, F2 and first generation backcrosses to both parental.

Maternity inference of the nine hybrid individuals was determined using a likelihood-based approach implemented in CERVUS 3.0^49^. Parentage inference was carried out using allele frequencies at the 51 microsatellite loci across all sable and roan individuals genotyped (hybrids were excluded). A threshold determined empirically by simulation (based in the log-likelihood ratio, LOD score) set the proportion of maternity tests that could be resolved with a strict confidence level of 90% and 99%. The number of candidate mothers was set at nine and the proportion of sampled candidate mothers was set at 90%. This simulates the chance that an unknown female may be the mother. We assumed that an average of 80% of loci per individual was typed and that an average of 1% of loci was mistyped. Critical LOD scores were calculated for the assignment of maternity to the nine females for the seven F1 hybrids. The same procedure was used to infer maternity of backcross individuals using as candidate mothers all the F1 females.

## Additional Information

**How to cite this article**: Vaz Pinto, P. *et al.* Hybridization following population collapse in a critically endangered antelope. *Sci. Rep.* **6**, 18788; doi: 10.1038/srep18788 (2016).

## Supplementary Material

Supplementary Information
