# Supplementary material for: Hybridization following population collapse in a critically endangered antelope
Source: Sci Rep. 2016 Jan 6;6:18788. doi: 10.1038/srep18788 (PMC4702127; doi:10.1038/srep18788)
Supplement: Supplementary Information [file srep18788-s1.doc]

**SUPPLEMENTARY FIGURES**

**Supplementary Figure 1 | Example of photos obtained from camera trapping during the study period**. (a) Pure sable females and F1 hybrid calves; (b) Territorial roan bull; (c) Several hybrids; (d) Dominant F1 hybrid bull; (e) Female F1 hybrid attending her calf; (f) Male and female backcrosses in the foreground, and two F1 females in the background. All photos were obtained from camera traps set by one of the authors (Pedro Vaz Pinto).


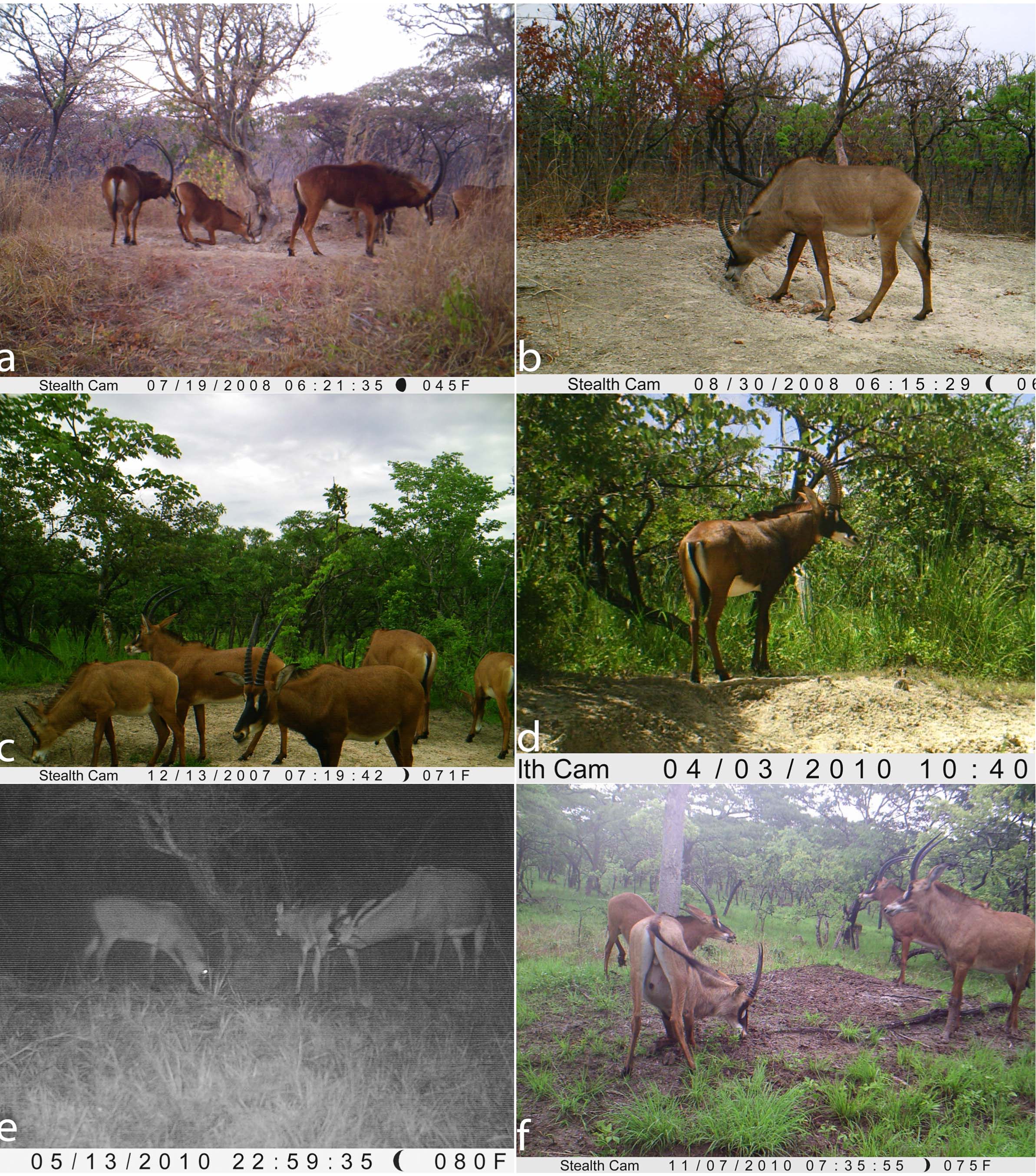


**Supplementary Figure 2 | Different stages of a giant sable translocation program to avoid local population extinction**. (a) Giant sable bull in Luando Nature Integral Reserve prior to translocation; (b) Female sable being offloaded by helicopter; (c) Female F1 hybrid marked and collared; (d) Giant sable bull with young females in temporary enclosure; (e) Translocated giant sable bull in Cangandala National Park with female in background; (f) Sable females and first pure calf born in Cangandala after the translocation operations. Photo 2a was taken by Sendi Lara Baptista, photo 2b was taken by Wolfram Brock, and photos 2c-f were taken by one of the authors (Pedro Vaz Pinto).


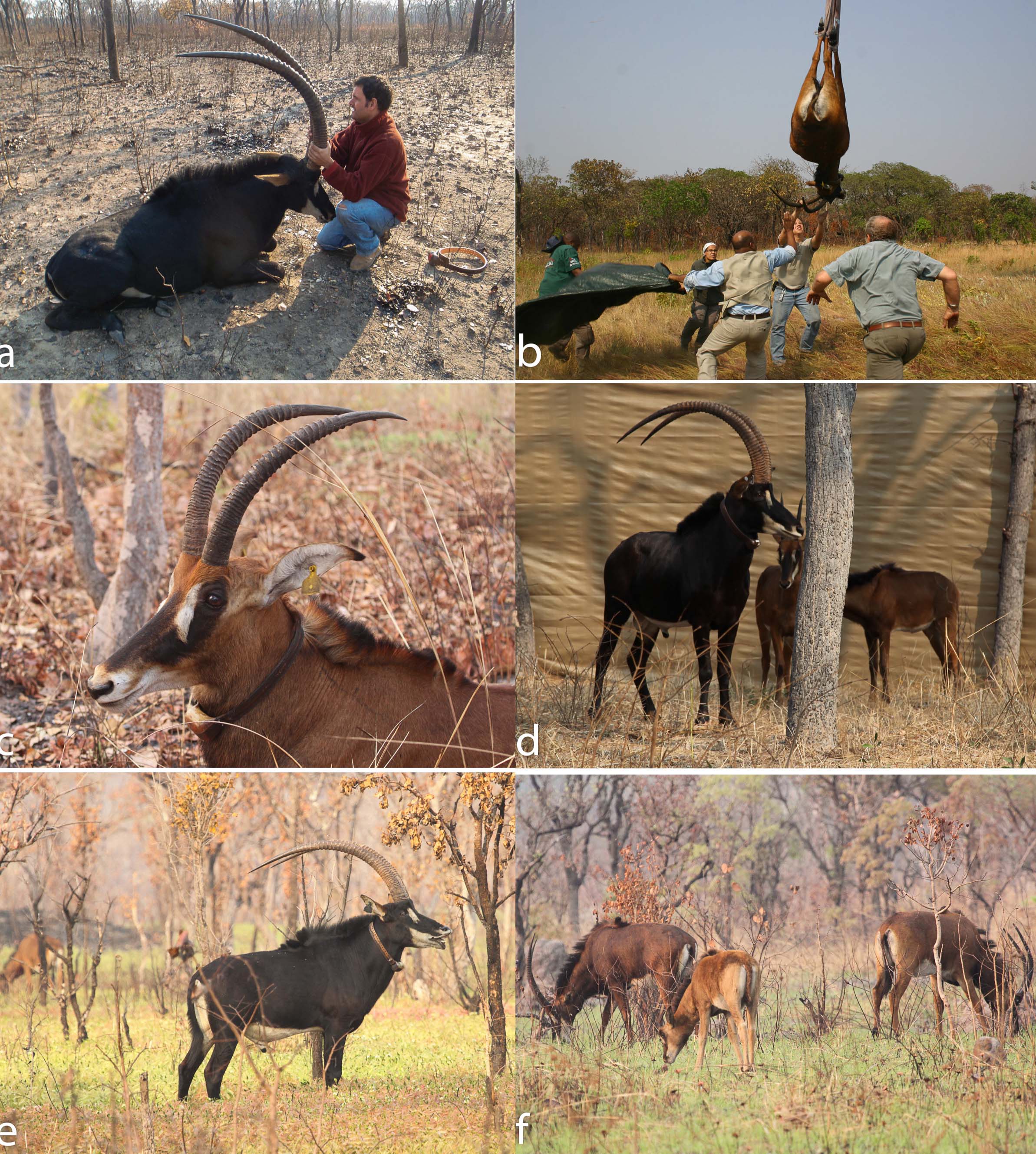


**SUPPLEMENTARY TABLES**

**Supplementary Table 1 | Quantification of camera-trapping data obtained during the study period.** Photos and video footage of sable, roan and their hybrids were obtained in Cangandala National Park through photographic and video cameras placed at natural salt licks (termite mounds) regularly visited by the animals. Access to this remote area involved major logistic difficulties, particularly during the first years, and so the number and type of cameras used and their operating periods varied greatly in space and over time.

|  | **Sable** | | |  | **Putative Hybrids** | | |  | **Roan** | | |
| --- | --- | --- | --- | --- | --- | --- | --- | --- | --- | --- | --- |
|  | **P** | **V** | **E** |  | **P** | **V** | **E** |  | **P** | **V** | **E** |
| **2005** | 19 | 0 | 2 |  | 4 | 0 | 1 |  | 0 | 0 | 0 |
| **2006** | 16 | 9782 | 12 |  | 3 | 7383 | 6 |  | 4 | 376 | 3 |
| **2007** | 1413 | 2773 | 10 |  | 639 | 3202 | 8 |  | 43 | 0 | 2 |
| **2008** | 2579 | 0 | 11 |  | 3068 | 0 | 12 |  | 1670 | 0 | 13 |
| **2009** | 1358* | 0 | 12 |  | 2534 | 0 | 21 |  | 629 | 0 | 16 |
| **2010** | 81* | 0 | 2 |  | 9856 | 0 | 46 |  | 1167 | 0 | 37 |
| **2011** | 2037 | 0 | 15 |  | 735 | 0 | 11 |  | 1707 | 0 | 34 |
| **TOTAL** | **7503** | **12555** | **64** |  | **16839** | **10585** | **105** |  | **5219** | **376** | **108** |

P, number of photos; V, seconds of video footage; E, number of independent events (i.e., visits by herd or individuals to salt licks in different days). * Between Sep 09 and Aug 10 pure sable were contained and without access to salt licks.

**Supplementary Table 2 | Inferred ancestry of sable x roan hybrids in Cangandala National Park, Angola.** Ancestry analysis was performed on nine individuals with phenotypically intermediate characteristics between sable and roan antelopes, which were born in Cangandala between 2003 and 2009. For each individual we estimated the maternal lineage based on mitochondrial DNA (mtDNA), and for the males the paternal lineage based on the SRY gene (Y). We used NEWHYBRIDS to estimate the posterior probability of assignment of each individual to sable, roan, their F1 and F2 hybrids, and the backcrosses to sable (BxSable) and roan (BxRoan). Maternity inference used a likelihood-based analysis implemented in CERVUS 3.0 and hybrids were ascribed to mothers with a strict confidence level of 99%.

| **Individual** | **Sex** | **Birth**  **year** | **mtDNA** | **Y** | **Inferred Ancestry** | | | | | |  |
| --- | --- | --- | --- | --- | --- | --- | --- | --- | --- | --- | --- |
|  |  |  |  |  | Sable | Roan | F1 | F2 | BxSable | BxRoan | Mother |
| ***HYB-1*** | F | 2003 | Sable | - | 0 | 0 | 1 | 0 | 0 | 0 | FEM-1 |
| ***HYB-2*** | F | 2004 | Sable | - | 0 | 0 | 1 | 0 | 0 | 0 | FEM-9 |
| ***HYB-3*** | F | 2004 | Sable | - | 0 | 0 | 1 | 0 | 0 | 0 | FEM-1 |
| ***HYB-4*** | M | 2006 | Sable | Roan | 0 | 0 | 1 | 0 | 0 | 0 | FEM-3 |
| ***HYB-5*** | M | 2006 | Sable | Roan | 0 | 0 | 1 | 0 | 0 | 0 | FEM-9 |
| ***HYB-6*** | F | 2007 | Sable | - | 0 | 0 | 0 | 0.006 | 0 | 0.994 | HYB-3 |
| ***HYB-7*** | F | 2007 | Sable | - | 0 | 0 | 1 | 0 | 0 | 0 | FEM-7 |
| ***HYB-8*** | F | 2008 | Sable | - | 0 | 0 | 1 | 0 | 0 | 0 | FEM-9 |
| ***HYB-9*** | M | 2009 | Sable | Roan | 0 | 0 | 0 | 0 | 0 | 1 | HYB-3 |

**Supplementary Table 3 | Genetic diversity measures for populations of sable *Hippotragus* *niger* and of roan *H. equinus* based on 51 microsatellite loci.** Values for Angola are presented for the overall sample and considering separately the Cangandala and Luando subpopulations. N: Number of individuals, Ho: observed heterozygosity; He: expected heterozygosity; Na: mean number of alleles per locus; and NPA: number of private alleles. Standard error values are given in parenthesis.

|  | N | Ho | He | Na | NPA |
| --- | --- | --- | --- | --- | --- |
| **Sable (overall)** | 57 | 0.360 (0.031) | 0.520 (0.036) | 4.31 (0.35) | 2.49 (0.28) |
| **Angola** | 35 | 0.309 (0.036) | 0.329 (0.037) | 2.47 (0.19) | 0.51 (0.12) |
| ***Cangandala*** | 9 | 0.253 (0.037) | 0.228 (0.032) | 1.82 (0.11) | 0.02 (0.02) |
| ***Luando*** | 26 | 0.330 (0.039) | 0.332 (0.038) | 2.45 (0.19) | 0.12 (0.05) |
| **Namibia** | 22 | 0.440 (0.038) | 0.450 (0.038) | 3.41 (0.27) | 1.06 (0.17) |
| **Roan (overall)** | 24 | 0.399 (0.043) | 0.488 (0.047) | 5.80 (0.64) | 3.98 (0.58) |
| **Angola** | 4 | 0.418 (0.053) | 0.492 (0.050) | 3.96 (0.25) | 0.61 (0.12) |
| **Namibia** | 5 | 0.495 (0.051) | 0.504 (0.050) | 3.26 (0.26) | 0.53 (0.13) |
| **South Africa** | 15 | 0.359 (0.044) | 0.396 (0.046) | 4.96 (0.42) | 1.02 (0.31) |
